# Molecular Mechanisms of Astaxanthin as a Potential Neurotherapeutic Agent

**DOI:** 10.3390/md19040201

**Published:** 2021-04-03

**Authors:** Eshak I. Bahbah, Sherief Ghozy, Mohamed S. Attia, Ahmed Negida, Talha Bin Emran, Saikat Mitra, Ghadeer M. Albadrani, Mohamed M. Abdel-Daim, Md. Sahab Uddin, Jesus Simal-Gandara

**Affiliations:** 1 Faculty of Medicine, Al-Azhar University, Damietta 34511, Egypt; isaacbahbah@gmail.com; 2 Faculty of Medicine, Mansoura University, Mansoura 35516, Egypt; sherief_ghozy@yahoo.com; 3 Department of Pharmaceutics, Faculty of Pharmacy, Zagazig University, Zagazig 44519, Egypt; mosalahnabet@gmail.com; 4 Faculty of Medicine, Zagazig University, Zagazig 44519, Egypt; ahmed.said.negida@gmail.com; 5 Department of Pharmacy, BGC Trust University Bangladesh, Chittagong 4381, Bangladesh; talhabmb@bgctub.ac.bd; 6 Department of Pharmacy, Faculty of Pharmacy, University of Dhaka, Dhaka 1000, Bangladesh; saikatmitradu@gmail.com; 7 Department of Biology, College of Science, Princess Nourah bint Abdulrahman University, Riyadh 11474, Saudi Arabia; gmalbadrani@pnu.edu.sa; 8 Pharmacology Department, Faculty of Veterinary Medicine, Suez Canal University, Ismailia 41522, Egypt; abdeldaim.m@vet.suez.edu.eg; 9 Department of Pharmacy, Southeast University, Dhaka 1213, Bangladesh; 10 Pharmakon Neuroscience Research Network, Dhaka 1207, Bangladesh; 11 Nutrition and Bromatology Group, Department of Analytical and Food Chemistry, Faculty of Food Science and Technology, University of Vigo—Ourense Campus, E32004 Ourense, Spain

**Keywords:** astaxanthin, neuroprotective agent, oxidative stress, neuroinflammation, neurological diseases

## Abstract

Neurological disorders are diseases of the central and peripheral nervous system that affect millions of people, and the numbers are rising gradually. In the pathogenesis of neurodegenerative diseases, the roles of many signaling pathways were elucidated; however, the exact pathophysiology of neurological disorders and possible effective therapeutics have not yet been precisely identified. This necessitates developing multi-target treatments, which would simultaneously modulate neuroinflammation, apoptosis, and oxidative stress. The present review aims to explore the potential therapeutic use of astaxanthin (ASX) in neurological and neuroinflammatory diseases. ASX, a member of the xanthophyll group, was found to be a promising therapeutic anti-inflammatory agent for many neurological disorders, including cerebral ischemia, Parkinson’s disease, Alzheimer’s disease, autism, and neuropathic pain. An effective drug delivery system of ASX should be developed and further tested by appropriate clinical trials.

## 1. Introduction

Marine carotenoids are highly antioxidant, reparative, antiproliferative, and anti-inflammatory and can be applied as photo-protective skin to inhibit harmful ultraviolet radiation effects [1,2]. Non-photosynthetic marine species are unable to produce carotenoids de novo, except for marine autotrophic organisms [3]. Several studies have already reported that marine animals may either accumulate carotenoids directly from food or partially modify them through the metabolic pathways [4,5]. Consequently, carotenoids obtained from several marine species act on various pathways, including the conversion of metal derivatives into harmless molecules, converting hydroperoxides into more stable compounds, acting as quenchers of singlet molecular oxygen, and preventing the formation of free radicals through the block of free radical oxidation reactions and inhibition of the auto-oxidation chain reaction [3,6,7]. 

Astaxanthin (ASX) is one of the marine carotenoids, which was originally isolated by Kuhn and Sorensen from a lobster [8]. ASX exists everywhere in nature; however, it particularly presents as a red-orange pigment in several marine animals, including salmonids, shrimp, and crayfish [9,10]. While plants, microbes, and microalgae may also produce ASX, the *Haematococcus pluvialis* chlorophyte algae are known to have the highest potential to accumulate ASX [11,12,13,14]. Nowadays, there are many synthetics ASX; nevertheless, health concerns have arisen concerning the use of synthetic ASX for medical purposes. ASX is closely related to other carotenoids, including zeaxanthin, lutein, and β-carotene; therefore, it shares many similar biological functions [3,15,16]. Previously, it has been reported that ASX is biologically more active than the aforementioned carotenoids [17,18,19]. ASX has been previously reported to have therapeutic anticancer, antidiabetic, anti-inflammatory, and antioxidant activities, and neuro-, cardiovascular, ocular, and skin-protective effects [20]. 

In terms of neurological protective effects, many studies have mentioned the role of ASX in neurological disorders, including cerebral ischemia, Parkinson’s disease (PD), Alzheimer’s disease (AD), autism, and neuropathic pain, which we will discuss in the following sections [21,22,23]. In this review, we aimed to explore the potential therapeutic use of ASX in neurological and neuroinflammatory diseases. 

## 2. Bioavailability and Pharmacokinetics of Astaxanthin

The administration of ASX with dietary oils, particularly fish oil, may promote the absorption of ASX and enhance the neutrophil’s phagocytic activity [19,24]. Studies showed enhanced bioavailability and antioxidant effects of ASX when administered alongside olive oil in rats [25,26]. Moreover, Otton and his colleagues [27] reported that ASX administration with fish oil reduced the production of nitric oxide (NO) and increased the release of calcium, superoxide dismutase (SOD), catalase, and glutathione peroxidase (GPx). Owing to the lipophilic nature of ASX, it was thought that ASX transforms metabolically in the rats’ tissues before it is extracted [28].

It was observed that a high-cholesterol diet might improve the absorption of ASX in humans, which is transported into the liver via the lymphatic system. Matrix dissolution and mixed micelles integration are two essential steps leading up to membrane absorption [24]. It should be incorporated with chylomicrons after absorbing it by intestinal mucosal cells to be transported to the liver. After that, ASX is integrated and transferred to the tissues by lipoproteins [29]. Okada et al. [30] reported that smoking could significantly reduce the half-life of ASX, indicating that smoking enhances the metabolism and elimination of ASX. This finding was confirmed by many investigators who demonstrated that the half-life of carotenoids is significantly affected by smoking [31,32]. The reported half-life of plasma ASX ranged between 16 and 21 h [28,33]. In terms of tolerability, Odeberg et al. [34] reported that a single dose of 40 mg for healthy volunteers was well-tolerated. 

## 3. Astaxanthin for Neurological Disorders

### 3.1. Alzheimer’s Disease

AD is a chronic and serious neurodegenerative disease characterized by impairment of memory and cognitive function. In recent decades, the prevalence of AD has risen significantly [35,36]. It may have a huge effect and obstacles on the well-being and the ability to lead a healthy life by the affected patients [37,38]. The excessive accumulation of β-amyloid protein (Aβ) in the cerebral cortex and hippocampus is one of AD’s main features [39]. Aβ contributes to oxidative stress production by forming reactive oxygen and nitrogen species [40]. Many adverse effects are related to oxidative stress production, including the formation of neurofibrillary tangles, inflammation, apoptosis, protein oxidation, and lipid peroxidation [41,42]. As a result of these disturbances, a reduction in cognitive functions can be developed in response to the significant damage of neural connections between the cerebral cortex and the hippocampus [43]. Many researchers have proposed antioxidants supplementation to prevent oxidative stress’ adverse effects by enhancing the endogenous oxidative defense [44,45,46]. Previous studies have demonstrated the potential effective role that ASX might have in the management of AD. A previous study by Taksima et al. [47], where the authors used ASX powder obtained from shrimp shells (*Litopenaeus vannamei*), showed that Wistar rats with AD had significantly improved levels of their cognitive abilities. ASX has significantly enhanced spatial and non-spatial memory and reduced neurodegeneration, assessed by the object recognition test and Aβ plaque level [47]. It was thought that ASX might improve GPx activity, which was observed to be suppressed due to mitochondrial dysfunction and Aβ accumulation [47,48]. 

Moreover, ASX participates in reducing protein carbonyl and malondialdehyde (MDA) levels, which result from the destruction of polyunsaturated fatty acids by the reactive oxygen species (ROS) and act on inducing neuronal deterioration [49,50]. Likewise, the role of ASX in the elimination of superoxide anion has been reported [51]. In AD, many reports have linked the production of ROS and neuronal death due to the formation of senile plaques [52,53]. Compared to the vehicle-AD group, it has demonstrated a significant reduction in hippocampal and cortical neuronal loss in the oral ASX group [47,54]. In the same context, Che et al. [55] reported that after application of synthesized ASX, their double transgenic mice (APP/PS1) showed improved cognitive abilities by reducing neuroinflammation and the related oxidative distress, which is a major cause that can inaugurate the mechanism and impact the prognosis of AD [56,57]. A study has shown that the number of references and working memory errors has significantly reduced in APP/PS1 treated with ASX. Moreover, ASX has improved the APP/PS1 behavior, reduced the hippocampal and cortical Aβ numbers, and decreased the soluble and insoluble Aβ 40 and Aβ 42 levels [55]. These changes were accompanied by a significant elevation in the level of superoxide dismutase (SOD) and a significant decline in the nitric oxide (NO) and nitric oxide synthase (NOS) levels. Interestingly, it was reported that ASX might induce a significant suppression of p-Tau expression; however, it did not affect the regulation of p-GSK-3β expression [58]. ASX possesses a powerful anti-inflammatory activity that abolishes the expression of inflammatory mediators, including TNF-α, PGE2, and IL-1β, and inhibits the development of nitric oxide (NO) as well as the NF-κB-dependent signaling pathway [36,59].

Other studies have described similar anti-inflammatory effects of astaxanthin via using different laboratory models. ASX, at a dose of 50 μM, declined the release of inflammatory mediators in activated microglial (BV-2 cell line) cells via the regulation of NF-κB cascade factors (e.g., p-IKKα, p-IκBα, and p-NF-κB p65, IL-6, and MAPK) [60]. 

In terms of cytokines, ASX sub-retinally reduced the level of TNF-α but not IL-1β [55,61]. Furthermore, ASX has been reported to be effective in terms of apoptosis suppression in APP/PS1 mice, as it suppresses the expression of caspase-9 and caspase-3 proteins [55]. The favorable effects of ASX in decreasing any potentially present oxidative stress are owed to the capability to pass the blood–brain barrier, enabling it to perform its favorable effects. The exact mechanism explaining the anti-inflammatory actions of ASX is not well understood. However, many studies have reported some observations that might help understand it. A previous investigation by Wang et al. [62] reported that ASX significantly reduced oxidative stress and reduced the present ischemia, which occurred secondary to brain injury. Via the ERK1/2 pathway, ASX also induced the expression of the Ho-1 enzyme (which has antioxidant properties), reducing cell death and protecting neuroblastoma cells that were susceptible to injury [62]. The favorable effects of ASX were also demonstrated by Wen et al. [63], that showed the neuroprotective role that this compound plays in the hippocampal HT22 cells of their mice also by increasing the expression of Ho-1 antioxidant activities. Another mechanism for enhancing the cognitive ability in rats with AD is the inhibition of glutathione-induced cell death, which has been previously reported to take part in the prognosis and AD severity [64,65]. Moreover, ASX demonstrated the protective effects on mitochondria’s double membrane system with boosting efficient energy production [9,66]. Specifically, ASX protected the mitochondria of cultured nerve cells from toxic attacks and increased mitochondrial activity through enhanced oxygen consumption without increased reactive oxygen species production [66,67,68], indicating its potential efficacy in the management and possible prevention of neurodegenerative diseases and neuroinflammation [9,69].

**Figure 1:**
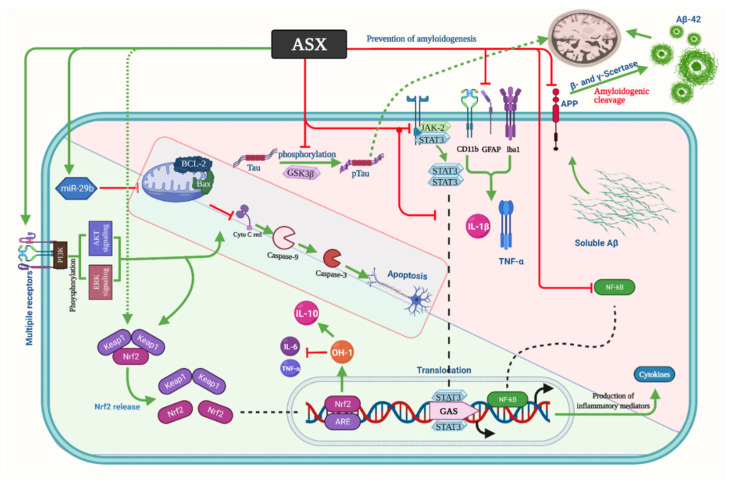
Astaxanthin mechanism of action in Alzheimer’s disease. Aβ: Amyloid beta, APP: β-amyloid precursor protein, ASX: Astaxanthin, NF-κB: Nuclear factor-kappa B, TNF-α: Tumor necrosis factor-alpha, IL: Interleukin, Iba1: Ionized calcium-binding adaptor molecule 1, GFAP: Glial fibrillary acidic protein, STAT3: Signal transducer and activator of transcription 3, JAK2: Janus Kinase 2, GSK3β: Glycogen synthase kinase 3 beta, p-Tau: Phosphorylated tau, Bcl-2: B-cell lymphoma 2, Bax: Bcl-2-associated X protein, Nrf2: Nuclear factor erythroid 2-related factor 2, GAS: Glyoxylate, anapleurotic and succinyl CoA, OH: Hydroxide, Keap1: Kelch-like ECH-associated protein 1, Akt: Protein kinase B.

Hongo et al. [58] used a new AD model, the App^NL-G-F^ mice model, which is associated with mild memory decline, microglial formation, increased level of p-Tau, and accumulation of Aβ_42_ in the hippocampus. Their findings indicated that ASX significantly reduced the Aβ_42_ deposition, p-Tau, and Iba1 fraction. On the other hand, it increased the glutathione biosynthesis, leading to an increase in the hippocampal parvalbumin-positive-positive neuron density, which plays a significant role in gamma oscillation production [70]. According to a recent study, gamma oscillations’ optogenetic or sensory activation led to the decline of Aβ peptides in the hippocampus of the AD mouse model (5XFAD mouse) due to microglial activation and the resulting increase in Aβ microglial uptake [71]. A reduction in the Iba1 fraction may be attributed to reducing Aβ42 precipitation in ASX-fed AppNL-G-F mice as microglia accumulate around Aβ deposition [72]. Regarding the effect of ASX on p-Tau, two pathways were suggested: the amyloid cascade theory and the autophagy-mediated degradation [73]. The p-Tau fraction was positively correlated with the Aβ42 fraction, which supports the amyloid cascade theory [58]. The promotion of nuclear factor erythroid 2-related factor 2 (Nrf2)/antioxidant response element (ARE) by ASX, resulting in reducing p-Tau, suggested the effect of ASX on the autophagy [74]. In AD-like model rats, which were induced using hydrated aluminum chloride (AlCl3.6H2O) solution, Hafez and her colleagues showed that ASX significantly reduced the disposition of Aβ1-42, the level of MDA, the activity of acetylcholinesterase and monoamine oxidase, and the expression of β-site amyloid precursor protein cleaving enzyme 1 (BACE1). Moreover, ASX significantly elevated the miRNA-124 expression, Nrf2 upregulation, and the content of serotonin and acetylcholine [75]. Figure 1 summarizes the aforementioned mechanisms of ASX in AD.

### 3.2. Parkinson’s Disease 

PD is the second most common neurodegenerative disorder [76]. It is age-related and is caused by oxidative stress and neuroinflammation [77]. The global prevalence of PD is estimated to be 0.1–0.2%, which increases with age (>80 years old) up to 3% [78,79]. PD occurs mainly due to the motor and non-motor dysfunctional disorders, which are attributable to loss of the dopaminergic neurons, the devastation of the non-dopaminergic ones, and the accumulation of the alpha-synuclein, which is the major component of Lewy bodies and plays a significant role in the development and progression of PD [80,81]. There are strong evidences that firstly, it affects the vagus nerve motor nucleus, the olfactory bulbs, and the nucleus, then the locus coeruleus, and thus, finally, the substantia nigra. Cortical regions of the brain at a later point are impaired. Damages to these particular neural structures are the result of numerous pathophysiological alterations that not only affect the engine system, but also neurological and neuropsychological systems [82]. Although many treatment modalities are currently approved for PD management, many adverse events have been associated, and therefore, many approaches have been made to discover novel multi-targeting modalities to treat PD properly. In the last decade, numerous miRNAs have been recognized and suggested as key gene expression regulators in human cells [83]. 

Almost all genes related to PD have been observed to be mediated by miRNAs, including alpha-synuclein (SNCA), LRRK2, and several transcription and growth factors [84]. MiR-7 was found to influence the SNCA accumulation and engaged with the PD etiology [85]. MiR-7 decreasing of the SN area was known as a therapeutic indicator of PD, not only involving SNCA accumulation but also dopaminergic neuron loss and miR-7 replacement therapy [86]. This was indicated by Shen et al. [87], who reported that ASX could decrease the previously induced stress in the endoplasmic reticulum by acting on the miR-7/SNCA axis to reduce the potential nerve damage that may be caused by PD. SNCA is the main gene that is usually responsible for the development and early initiation of PD. During the initiation and development of multiple neurodegenerative disorders like PD, miRNAs are presented spatially and temporally, suggesting that miRNAs play a key role in PD pathogenesis. In vivo, they also found that ASX has a potential protective effect against the neuron injury induced by 1-methyl-4-phenyl-1,2,3,6-tetrahydropyridine (MPTP) via a miR-7/SNCA axis. On the other hand, the favorable events of ASX were not reported in the animal study by Grimmig et al. [88] that reported that the compound’s efficacy was limited in aged animals with PD as it was not able to counteract the toxicity of MPTP. However, they found that in both young and aged mice, the neuronal damage in the substantial nigra was prevented by ASX. Therefore, they suggested that any clinical recommendations for PD should take aging as an important factor. Previous studies have investigated the potential effects that modified ASX compounds might have on PD. These compounds include the docosahexaenoic acid (DHA)-acylated ASX ester and ASX in combination with the non-esterified ASX and DHA. 

**Figure 2:**
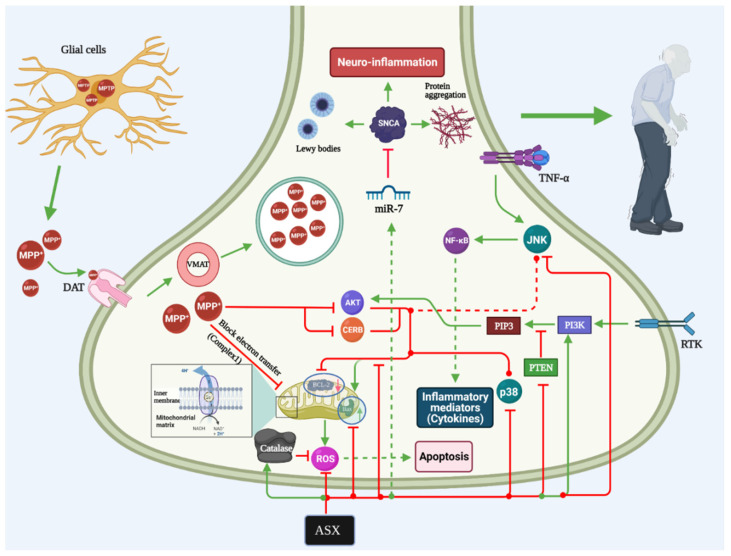
Astaxanthin mechanism of action in Parkinson’s disease. NF-κB: Nuclear factor-kappa B, TNF-α: Tumor necrosis factor-alpha, Akt: Protein kinase B, ASX: Astaxanthin, ROS: Reactive oxygen species, RTK: Receptor tyrosine kinase, PIP3: Phosphatidylinositol-3,4,5-triphosphate, PI3K: Phosphatidylinositol 3‑kinase, JNK: c-Jun N-terminal kinase, CREB: cAMP Response Element-Binding Protein, PTEN: Phosphatase and tensin homolog deleted on chromosome 10, MPTP: 1-methyl-4-phenyl-1,2,3,6-tetrahydropyridine, VMAT: Vesicular monoamine transporter, DAT: Dopamine transporter, SNCA: Alpha-synuclein.

Evidence shows that the first compound’s efficacy was significantly better than the latter one in reducing the development of MPTP-induced PD in mice [89]. Wang et al. [89] also proved that DHA-ASX could significantly reduce the progression of PD by reducing the apoptotic phenomena of the dopamine neurons by acting through the P38 MAPK and JNK pathway (Figure 2). Although the three ASX-derived compounds showed favorable events in reducing oxidative stress, DHA-ASX was the only significant compound that can limit PD progression by reducing cell apoptosis. A previous study also indicated ASX’s ability to inhibit the activities of the mitogen-activated protein kinase and P13K/AKT, which might favor its actions on many neurological diseases, such as PD [90]. Moreover, it has been indicated that ASX also has anti-oxidative stress that is attributable to MPP mechanisms in PC12 cells by acting through the NOX2/HO-1 and NR1/SP1 pathways [91,92]. Previous studies indicated the favorable events of ASX that showed that ASX administration is associated with decreased reactive oxygen species synthesis, reduced mitochondrial dysfunction, and reduced cellular apoptosis [93,94].

### 3.3. Neuropathic Pain and Central Nervous System Injuries

Neuropathic pain develops when a disorder or an injury occurs within the somatosensory pathway, stimulating the underlying affected neurons [95]. Neuropathic pain development was previously explained by many mechanisms and pathways, mainly dependent on the effector mediator. Many inflammatory mediators, such as prostaglandins, cytokines, and reactive oxygen species, in addition to the neuromodulators, which mainly include glutamate, have been frequently observed in such painful events [96,97,98,99]. These factors can cause pain through apoptosis, neuron firing, and impacting many structures and processes, such as microglia, astrocytes, and ion currents [100]. Although many treatment modalities can be used to manage neuropathic pain, approaching to obtain favorable modalities that may have more advantages is essential to enhance the quality of care. One of the treatment modalities that has shown successful results recently is counteracting the neuroinflammatory process. Gugliandolo et al. [101] found that reversing the neuroinflammation was protective against peripheral nerve injury and neuropathic pain in an experimental study. In terms of experimental studies on ASX, Keudo et al. [51] reported that favorable effects of reducing pain in carrageenan-induced pain and edema in mice were significantly associated with ASX that was also obtained from *Litopenaeus vannamei* and was efficacious in reducing the painful sensations and inflammation. Sharma et al. [102] supported this by concluding that ASX reduced the oxidative stress that resulted in behavioral and chemical alternations in vivo and in vitro experiments, where the objects suffered from induced neuropathic pain. 

Moreover, the effective anti-inflammatory effects of ASX were further proven by its ability to reduce chronic pain by reducing the potential thermal hyperalgesia and the possible presence of depressive symptoms in the affected mice [103]. Another report by Fakhri et al. [104] showed that ASX is able to significantly inhibit ERK1/2 and activate protein kinase B (AKT), which, in turn, are responsible for initiating chemical and thermal painful sensations. Another potential mechanism of ASX actions is that it blocks the inflammatory signaling and reduces the associated mediators as glutamatergic-phospo-p38-mitogen-activated protein kinase (p-p38MAPK) and NR2B [105]. Long-standing exposure of neurons to glutamate contributes to cell death [106]. There are many adverse effects attributed to the neuronal exposure to glutamate, including neuronal damage triggered by L-glutamate, retinal ganglion cells death due to glutamate stress, and cytotoxicity of HT22 cells, which is mediated by mitochondrial dysfunction, inactivation of caspase, and dysregulation of the AKT/GSK-3b signaling pathway [107,108,109,110]. Fortunately, ASX provides neuroprotective effects against all of these adverse effects. In cases of spinal cord injury (SCI), it is known that NMDARs subunits like NMDARs 2B (NR2B) and glutamate participate in the neuropathy pain pathway [99,111]. NR2B is a cation channel that is essential for many forms of synaptic plasticity and mediates the neurotransmission of glutamate and many other aspects of development and synaptic transmission in neuropathy pain [112].

**Figure 3:**
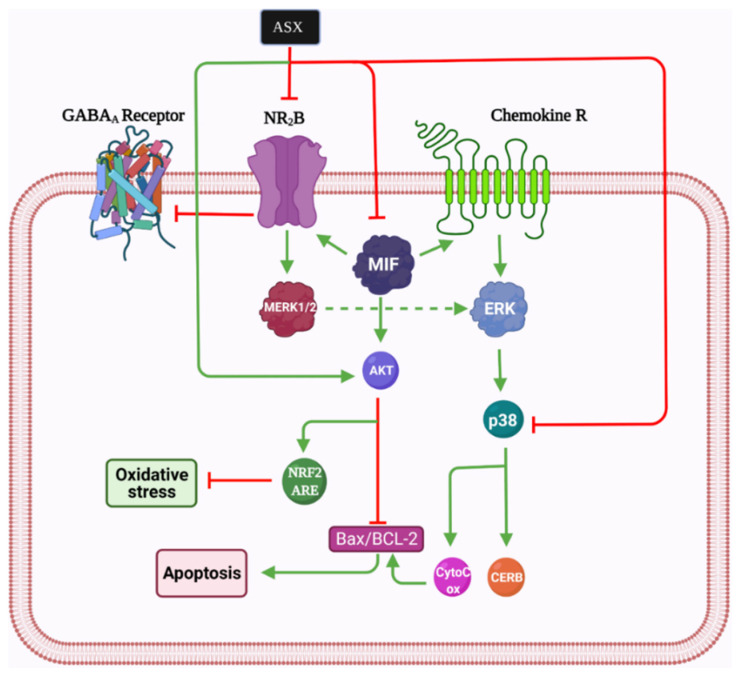
Astaxanthin mechanism of action in neuropathic pain. Bcl-2: B-cell lymphoma 2, Bax: Bcl-2-associated X protein, Nrf2: Nuclear factor erythroid 2-related factor 2, Akt: Protein kinase B, ASX: astaxanthin, CREB: cAMP Response element-binding protein, MERK: Mitogen-extracellular signal-regulated kinases, MIF: Macrophage migration inhibitory factor, NR2B: N-methyl D-aspartate receptor subtype 2B, GABA: Gamma-aminobutyric acid.

However, NR2B activation can be toxic for the spinal cord. It has been proposed that ASX participates in reducing neuropathic pain by inhibiting the glutamate-initiated signaling pathway through decreasing the expression of NR2B and p-p38MAPK [2,105,113]. Moreover, ASX inhibits the MIF, p-p38MAPK, p-ERK, and AKT pathways and stimulates the p-AKT and ERK pathways [114]. MIF upregulates NR2B; therefore, it can be considered a major mediator of neuropathic pain, and it has been shown by several cell lines in the peripheral and central nervous system, especially within cells located in sensory transmission regions [115]. Furthermore, in response to tissue damage and stress, it is dramatically elevated, often reaching concentrations about 1000 times higher than other cytokines causing pain [116]. In general, in view of its antioxidant, anti-inflammation, and anti-apoptotic mechanisms, ASX may be considered a new prospect for lowering neuropathic pain in animal models. The reduction of NR2B and MIF, which are very significant in the occurrence of neuropathic pain after SCI, may be partly involved (Figure 3).

### 3.4. Autism

The prevalence of autism has recently increased, with many social, behavioral, and communicational burdens over the affected patients and the surrounding individuals [117,118,119]. In addition to having many neurodegenerative events being involved in its mechanism [120,121,122], autism is also associated with increased levels and frequencies of synthesis and release of various proinflammatory mediators [123]. Gastrointestinal (GI) symptoms are common among autism patients. The gut microbiota regulates neuropsychological functions, intestinal homeostasis, and functional GI disturbances through the microbiota-gut-brain axis [124]. Moreover, previous studies have suggested that patients with autism might have an underlying degree of oxidative stress [125,126,127,128]. Consequently, previous studies have demonstrated that ASX might have a potential role in reducing the inflammatory state and oxidative stress that might be present in autistic patients [129,130]. Furthermore, it was believed that ASX could significantly reduce bacterial loads and attenuate gastric inflammation in mice infected with H. pylori, and increase the production of IgA antibody-secreting cells in the small intestine of mice. Therefore, ASX could have a potential in the prevention or treatment of dysbiosis and its associated diseases like autism, AD, and PD [131].

Fernández et al. [132] previously suggested the administration of carotenoids as routine food in patients with autism to reduce the potential oxidative stress and inflammatory state. Al-Amin et al. [133] also reported that ASX reduced the actions of catalase activities, restricted lipid peroxidation, and reduced the levels of nitric oxide, which are involved in developing oxidative stress. This has led to a significant enhancement in the assessed behavioral parameters and a significant increase in the assessed paw withdrawal latency in the studied mice that suffered from autism, secondary to valproic-acid induction [133].

### 3.5. Cerebral Ischemia

Prolonged cerebral ischemia can lead to the development of irreversible adverse events. Previous investigations demonstrated a potential impact of ASX carotenoid for reducing the severity of cerebral ischemia and potentiating the chances of brain tissue recovery. Xue et al. [134] reported that ASX was significantly able to reduce ischemia and improve the cognitive and learning abilities in their model of mice that were subjected to repeated cerebral ischemia by reducing apoptosis and hippocampal damage. Some mechanisms can explain the prevention of brain disorders by ASX by enhancing reperfusion rates following ischemia. These include activation of the Nrf2–ARE pathway, reducing the reactive oxygen species levels, reducing apoptosis, and enhancing nerve regeneration [135]. 

Moreover, evidence shows that ASX possesses an essential role in providing the necessary oxygenation for the apoptotic brain tissue through the GSK3β/PI3K/Nrf2/Akt pathways [136]. Wang et al. [135] confirmed this by indicating that ASX was able to enhance the prognosis and motor functions through the cAMP/protein kinase A (PKA)/cAMP response element-binding protein (CREB). Previous studies also showed that ASX has protective roles in acute cerebral infarctions and brain injury [137,138].

## 4. Potential of Astaxanthin in Counteracting Neuroinflammation

A huge body of literature supports the role of ASX in preventing neuroinflammation, which makes it a potential candidate for further testing in various neurological disorders, where neuroinflammation plays a key role in disease pathology and progression, including AD, PD, nerve injury, cerebral ischemia, and autism. 

For example, Che et al. [55] reported improved cognitive abilities in AD transgenic mice by reducing neuroinflammation and the related oxidative distress [56,57], and Kidd et al. [9] reported similar favorable results on the mitochondria and microcirculation [69]. Gugliandolo et al. [101] also reported that reversing the neuroinflammation was effective in protecting against peripheral nerve injury and neuropathic pain. Similarly, counteracting the neuroinflammation has recently been shown to improve recovery in Parkinson’s disease experimental models [139]. Impellizzeri et al. also reported that reversing the neuroinflammation was an alternative strategy for the treatment of cerebral ischemia and particularly for vascular dementia. 

Based on the aforementioned ASX mechanisms of actions, the promising findings in experimental studies, and the fact that neuroinflammation plays a key role in AD, PD, nerve injury, cerebral ischemia, and autism, we support the advancement of this neurotherapeutic candidate to further testing in clinical trials.

## 5. Safety of Astaxanthin

Many studies reported that ASX is safe and has no side effects or toxic effects when accumulating in animal or human tissues [26]. However, excessive consumption of ASX may lead to altering the pigmentation of the skin of animals [24]. The accumulation of ASX was also observed in the eyes of the rats [140]. Administration of ASX was associated with increased antioxidant enzymes and reduced blood pressure in hypertensive rats [141]. As a feed additive, the United States Food and Drug Administration (FDA) approved ASX at up to 80 mg/kg, while the European Food Safety Authority (EFSA) approved up to 100 mg/kg [142]. 

In terms of daily intake, it was reported that 0.034 mg/kg/day of natural ASX is the acceptable daily intake in humans [143]. However, recent clinical trials reported favorable outcomes with higher doses up to 8 mg per day or even higher [144,145]. In a safety report, the investigators have assessed more than 80 clinical trials to detect the side effects and safety concerns of ASX [146]. Their findings highlighted that there were no serious adverse effects reported in any one of the evaluated studies, even in the studies that administrated high doses of ASX (up to 45 mg) [147]. Some mild adverse events such as increased frequency of bowel movements were reported [148]. Moreover, there was no detectable change in the liver parameters [149].

## 6. Conclusions

ASX, a ketocarotenoid extracted from marine carotenoids, provides various health benefits in a wide variety of diseases. As a multi-target neuroprotective agent, ASX tackles neurodegenerative diseases’ pathophysiology through antioxidant, anti-inflammatory, and anti-apoptotic mechanisms. Moreover, through its fat-soluble properties, ASX would be able to effectively pass through the blood–brain barrier. Therefore, ASX seems to be an excellent candidate for more evaluation of the neuroprotective properties, which would eventually result in ASX becoming a novel neurotherapeutic agent. Although the current evidence supports the neuroprotective pharmacological effects of ASX, there is a lack of an effective drug delivery system in the previous studies. Therefore, future clinical trials should be conducted to examine the possible delivery methods. Moreover, there is a need to further investigate the precise pathophysiological pathways involved in neurodegeneration and the possible neuroprotective mechanisms of ASX in humans.

## References

[1] Maoka T. (2011). Carotenoids in marine animals. Mar. Drugs.

[2] Attal N., Cruccu G., Baron R., Haanpää M., Hansson P., Jensen T.S., Nurmikko T. (2010). EFNS guidelines on the pharmacological treatment of neuropathic pain: 2010 revision. Eur. J. Neurol..

[3] Galasso C., Corinaldesi C., Sansone C. (2017). Carotenoids from Marine Organisms: Biological Functions and Industrial Applications. Antioxidants.

[4] Chuyen H.V., Eun J.B. (2017). Marine carotenoids: Bioactivities and potential benefits to human health. Crit. Rev. Food Sci. Nutr..

[5] Maoka T., Akimoto N., Tsushima M., Komemushi S., Mezaki T., Iwase F., Takahashi Y., Sameshima N., Mori M., Sakagami Y. (2011). Carotenoids in marine invertebrates living along the Kuroshio current coast. Mar. Drugs.

[6] Brotosudarmo T.H.P., Limantara L., Setiyono E., Heriyanto (2020). Structures of Astaxanthin and Their Consequences for Therapeutic Application. Int. J. Food Sci..

[7] Phaniendra A., Jestadi D.B., Periyasamy L. (2015). Free radicals: Properties, sources, targets, and their implication in various diseases. Indian J. Clin. Biochem. IJCB.

[8] Davinelli S., Nielsen M.E., Scapagnini G. (2018). Astaxanthin in Skin Health, Repair, and Disease: A Comprehensive Review. Nutrients.

[9] Kidd P. (2011). Astaxanthin, cell membrane nutrient with diverse clinical benefits and anti-aging potential. Altern. Med. Rev. A J. Clin. Ther..

[10] Mezzomo N., Ferreira S.R.S. (2016). Carotenoids Functionality, Sources, and Processing by Supercritical Technology: A Review. J. Chem..

[11] Zhang C., Chen X., Too H.P. (2020). Microbial astaxanthin biosynthesis: Recent achievements, challenges, and commercialization outlook. Appl. Microbiol. Biotechnol..

[12] Mularczyk M., Michalak I., Marycz K. (2020). Astaxanthin and other Nutrients from *Haematococcus pluvialis*-Multifunctional Applications. Mar. Drugs.

[13] Khoo K.S., Lee S.Y., Ooi C.W., Fu X., Miao X., Ling T.C., Show P.L. (2019). Recent advances in biorefinery of astaxanthin from Haematococcus pluvialis. Bioresour. Technol..

[14] Shah M.M., Liang Y., Cheng J.J., Daroch M. (2016). Astaxanthin-Producing Green Microalga Haematococcus pluvialis: From Single Cell to High Value Commercial Products. Front. Plant Sci..

[15] Higuera-Ciapara I., Félix-Valenzuela L., Goycoolea F.M. (2006). Astaxanthin: A review of its chemistry and applications. Crit. Rev. Food Sci. Nutr..

[16] Martín J.F., Gudiña E., Barredo J.L. (2008). Conversion of beta-carotene into astaxanthin: Two separate enzymes or a bifunctional hydroxylase-ketolase protein? Microb. Cell Factories.

[17] Sztretye M., Dienes B., Gönczi M., Czirják T., Csernoch L., Dux L., Szentesi P., Keller-Pintér A. (2019). Astaxanthin: A Potential Mitochondrial-Targeted Antioxidant Treatment in Diseases and with Aging. Oxid. Med. Cell. Longev..

[18] Fakhri S., Abbaszadeh F., Dargahi L., Jorjani M. (2018). Astaxanthin: A mechanistic review on its biological activities and health benefits. Pharmacol. Res..

[19] Ambati R.R., Phang S.M., Ravi S., Aswathanarayana R.G. (2014). Astaxanthin: Sources, extraction, stability, biological activities and its commercial applications--a review. Mar. Drugs.

[20] Yuan J.P., Peng J., Yin K., Wang J.H. (2011). Potential health-promoting effects of astaxanthin: A high-value carotenoid mostly from microalgae. Mol. Nutr. Food Res..

[21] Wang M., Deng X., Xie Y., Chen Y. (2020). Astaxanthin Attenuates Neuroinflammation in Status Epilepticus Rats by Regulating the ATP-P2X7R Signal. Drug Des. Dev. Ther..

[22] Xu L., Zhu J., Yin W., Ding X. (2015). Astaxanthin improves cognitive deficits from oxidative stress, nitric oxide synthase and inflammation through upregulation of PI3K/Akt in diabetes rat. Int. J. Clin. Exp. Pathol..

[23] Lu Y., Wang X., Feng J., Xie T., Si P., Wang W. (2019). Neuroprotective effect of astaxanthin on newborn rats exposed to prenatal maternal seizures. Brain Res. Bull..

[24] Barros M.P., Marin D.P., Bolin A.P., de Cássia Santos Macedo R., Campoio T.R., Fineto C., Guerra B.A., Polotow T.G., Vardaris C., Mattei R. (2012). Combined astaxanthin and fish oil supplementation improves glutathione-based redox balance in rat plasma and neutrophils. Chem. Biol. Interact..

[25] Ranga Rao A., Raghunath Reddy R.L., Baskaran V., Sarada R., Ravishankar G.A. (2010). Characterization of microalgal carotenoids by mass spectrometry and their bioavailability and antioxidant properties elucidated in rat model. J. Agric. Food Chem..

[26] Rao A.R., Sindhuja H.N., Dharmesh S.M., Sankar K.U., Sarada R., Ravishankar G.A. (2013). Effective inhibition of skin cancer, tyrosinase, and antioxidative properties by astaxanthin and astaxanthin esters from the green alga Haematococcus pluvialis. J. Agric. Food Chem..

[27] Otton R., Marin D.P., Bolin A.P., De Cássia Santos Macedo R., Campoio T.R., Fineto C., Guerra B.A., Leite J.R., Barros M.P., Mattei R. (2012). Combined fish oil and astaxanthin supplementation modulates rat lymphocyte function. Eur. J. Nutr..

[28] Page G.I., Davies S.J. (2002). Astaxanthin and canthaxanthin do not induce liver or kidney xenobiotic-metabolizing enzymes in rainbow trout (Oncorhynchus mykiss Walbaum). Comp. Biochem. Physiology. Toxicol. Pharmacol. CBP.

[29] Olson J.A. (1994). Absorption, transport and metabolism of carotenoids in humans. Pure Appl. Chem..

[30] Okada Y., Ishikura M., Maoka T. (2009). Bioavailability of astaxanthin in Haematococcus algal extract: The effects of timing of diet and smoking habits. Biosci. Biotechnol. Biochem..

[31] Kelly G.S. (2002). The interaction of cigarette smoking and antioxidants. Part I: Diet and carotenoids. Altern. Med. Rev. A J. Clin. Ther..

[32] Kvaavik E., Totland T.H., Bastani N., Kjøllesdal M.K., Tell G.S., Andersen L.F. (2014). Do smoking and fruit and vegetable intake mediate the association between socio-economic status and plasma carotenoids? Eur. J. Public Health.

[33] Østerlie M., Bjerkeng B., Liaaen-Jensen S. (2000). Plasma appearance and distribution of astaxanthin E/Z and R/S isomers in plasma lipoproteins of men after single dose administration of astaxanthin. J. Nutr. Biochem..

[34] Mercke Odeberg J., Lignell A., Pettersson A., Höglund P. (2003). Oral bioavailability of the antioxidant astaxanthin in humans is enhanced by incorporation of lipid based formulations. Eur. J. Pharm. Sci. Off. J. Eur. Fed. Pharm. Sci..

[35] Karlawish J., Jack C.R., Rocca W.A., Snyder H.M., Carrillo M.C. (2017). Alzheimer’s disease: The next frontier-Special Report. Alzheimer’s Dement..

[36] Uddin M.S., Kabir M..T., Rahman M..S., Behl T., Jeandet P., Ashraf G.M., Najda A., Bin-Jumah M.N., El-Seedi H.R., Abdel-Daim M.M. (2020). Revisiting the Amyloid Cascade Hypothesis: From Anti-Aβ Therapeutics to Auspicious New Ways for Alzheimer’s Disease. Int. J. Mol. Sci..

[37] Sayed A., Bahbah E.I., Kamel S., Barreto G.E., Ashraf G.M., Elfil M. (2020). The neutrophil-to-lymphocyte ratio in Alzheimer’s disease: Current understanding and potential applications. J. Neuroimmunol..

[38] Bahbah E.I., Fathy S., Negida A. (2019). Is Alzheimer’s disease linked to Herpes simplex virus type 1 infection? A mini-review of the molecular correlation and the possible disease connections. Clin. Exp. Neuroimmunol..

[39] Uddin M.S., Kabir M.T., Tewari D., Mamun A.A., Mathew B., Aleya L., Barreto G.E., Bin-Jumah M.N., Abdel-Daim M.M., Ashraf G.M. (2020). Revisiting the role of brain and peripheral Aβ in the pathogenesis of Alzheimer’s disease. J. Neurol. Sci..

[40] Butterfield D.A., Swomley A.M., Sultana R. (2013). Amyloid β-peptide (1-42)-induced oxidative stress in Alzheimer disease: Importance in disease pathogenesis and progression. Antioxid. Redox Signal..

[41] Elhelaly A.E., AlBasher G., Alfarraj S., Almeer R., Bahbah E.I., Fouda M.M.A., Bungau S.G., Aleya L., Abdel-Daim M.M. (2019). Protective effects of hesperidin and diosmin against acrylamide-induced liver, kidney, and brain oxidative damage in rats. Environ. Sci. Pollut Res. Int..

[42] Abdel-Daim M.M., Abushouk A.I., Bahbah E.I., Bungau S.G., Alyousif M.S., Aleya L., Alkahtani S. (2020). Fucoidan protects against subacute diazinon-induced oxidative damage in cardiac, hepatic, and renal tissues. Env. Sci. Pollut. Res. Int..

[43] Bui T.T., Nguyen T.H. (2017). Natural product for the treatment of Alzheimer’s disease. J. Basic Clin. Physiol. Pharmacol..

[44] Nakajima A., Ohizumi Y. (2019). Potential Benefits of Nobiletin, A Citrus Flavonoid, against Alzheimer’s Disease and Parkinson’s Disease. Int. J. Mol. Sci..

[45] Hatziagapiou K., Kakouri E., Lambrou G.I., Bethanis K., Tarantilis P.A. (2019). Antioxidant Properties of Crocus Sativus L. and Its Constituents and Relevance to Neurodegenerative Diseases; Focus on Alzheimer’s and Parkinson’s Disease. Curr. Neuropharmacol..

[46] Wojsiat J., Zoltowska K.M., Laskowska-Kaszub K., Wojda U. (2018). Oxidant/Antioxidant Imbalance in Alzheimer’s Disease: Therapeutic and Diagnostic Prospects. Oxidative Med. Cell. Longev..

[47] Taksima T., Chonpathompikunlert P., Sroyraya M., Hutamekalin P., Limpawattana M., Klaypradit W. (2019). Effects of Astaxanthin from Shrimp Shell on Oxidative Stress and Behavior in Animal Model of Alzheimer’s Disease. Mar. Drugs.

[48] Shichiri M. (2014). The role of lipid peroxidation in neurological disorders. J. Clin. Biochem. Nutr..

[49] Hritcu L., Noumedem J.A., Cioanca O., Hancianu M., Kuete V., Mihasan M. (2014). Methanolic extract of Piper nigrum fruits improves memory impairment by decreasing brain oxidative stress in amyloid beta(1-42) rat model of Alzheimer’s disease. Cell. Mol. Neurobiol..

[50] Dalle-Donne I., Rossi R., Giustarini D., Milzani A., Colombo R. (2003). Protein carbonyl groups as biomarkers of oxidative stress. Clin. Chim. Acta Int. J. Clin. Chem..

[51] Kuedo Z., Sangsuriyawong A., Klaypradit W., Tipmanee V., Chonpathompikunlert P. (2016). Effects of Astaxanthin from Litopenaeus Vannamei on Carrageenan-Induced Edema and Pain Behavior in Mice. Molecules.

[52] Zhang Y.Y., Fan Y.C., Wang M., Wang D., Li X.H. (2013). Atorvastatin attenuates the production of IL-1β, IL-6, and TNF-α in the hippocampus of an amyloid β1-42-induced rat model of Alzheimer’s disease. Clin. Interv. Aging.

[53] Asadbegi M., Yaghmaei P., Salehi I., Komaki A., Ebrahim-Habibi A. (2017). Investigation of thymol effect on learning and memory impairment induced by intrahippocampal injection of amyloid beta peptide in high fat diet- fed rats. Metab. Brain Dis..

[54] Rahman S.O., Panda B.P., Parvez S., Kaundal M., Hussain S., Akhtar M., Najmi A.K. (2019). Neuroprotective role of astaxanthin in hippocampal insulin resistance induced by Aβ peptides in animal model of Alzheimer’s disease. Biomed. Pharmacother. Biomed. Pharmacother..

[55] Che H., Li Q., Zhang T., Wang D., Yang L., Xu J., Yanagita T., Xue C., Chang Y., Wang Y. (2018). Effects of Astaxanthin and Docosahexaenoic-Acid-Acylated Astaxanthin on Alzheimer’s Disease in APP/PS1 Double-Transgenic Mice. J. Agric. Food Chem..

[56] Kim H.A., Miller A.A., Drummond G.R., Thrift A.G., Arumugam T.V., Phan T.G., Srikanth V.K., Sobey C.G. (2012). Vascular cognitive impairment and Alzheimer’s disease: Role of cerebral hypoperfusion and oxidative stress. Naunyn-Schmiedeberg’s Arch. Pharmacol..

[57] Padurariu M., Ciobica A., Lefter R., Serban I.L., Stefanescu C., Chirita R. (2013). The oxidative stress hypothesis in Alzheimer’s disease. Psychiatr. Danub..

[58] Hongo N., Takamura Y., Nishimaru H., Matsumoto J., Tobe K., Saito T., Saido T.C., Nishijo H. (2020). Astaxanthin Ameliorated Parvalbumin-Positive Neuron Deficits and Alzheimer’s Disease-Related Pathological Progression in the Hippocampus of App(NL-G-F/NL-G-F) Mice. Front. Pharmacol..

[59] Solomonov Y., Hadad N., Levy R. (2018). The Combined Anti-Inflammatory Effect of Astaxanthin, Lyc-O-Mato and Carnosic Acid In Vitro and In Vivo in a Mouse Model of Peritonitis. J. Nutr. Food Sci..

[60] Kim Y.H., Koh Hk Fau-Kim D.-S., Kim D.S. (2010). Down-regulation of IL-6 production by astaxanthin via ERK-, MSK-, and NF-κB-mediated signals in activated microglia. Int. Immunopharmacol..

[61] Landon R., Gueguen V., Petite H., Letourneur D., Pavon-Djavid G., Anagnostou F. (2020). Impact of Astaxanthin on Diabetes Pathogenesis and Chronic Complications. Mar. Drugs.

[62] Wang H.Q., Sun X.B., Xu Y.X., Zhao H., Zhu Q.Y., Zhu C.Q. (2010). Astaxanthin upregulates heme oxygenase-1 expression through ERK1/2 pathway and its protective effect against beta-amyloid-induced cytotoxicity in SH-SY5Y cells. Brain Res..

[63] Wen X., Huang A., Hu J., Zhong Z., Liu Y., Li Z., Pan X., Liu Z. (2015). Neuroprotective effect of astaxanthin against glutamate-induced cytotoxicity in HT22 cells: Involvement of the Akt/GSK-3β pathway. Neuroscience.

[64] Kim G.H., Kim J.E., Rhie S.J., Yoon S. (2015). The Role of Oxidative Stress in Neurodegenerative Diseases. Exp. Neurobiol..

[65] Zhang Y., Wang W., Hao C., Mao X., Zhang L. (2015). Astaxanthin protects PC12 cells from glutamate-induced neurotoxicity through multiple signaling pathways. J. Funct. Foods.

[66] Wolf A.M., Asoh S., Hiranuma H., Ohsawa I., Iio K., Satou A., Ishikura M., Ohta S. (2010). Astaxanthin protects mitochondrial redox state and functional integrity against oxidative stress. J. Nutr. Biochem..

[67] Lu Y.P., Liu S.Y., Sun H., Wu X.M., Li J.J., Zhu L. (2010). Neuroprotective effect of astaxanthin on H(2)O(2)-induced neurotoxicity in vitro and on focal cerebral ischemia in vivo. Brain Res..

[68] Kim J.H., Choi W., Lee J.H., Jeon S.J., Choi Y.H., Kim B.W., Chang H.I., Nam S.W. (2009). Astaxanthin inhibits H2O2-mediated apoptotic cell death in mouse neural progenitor cells via modulation of P38 and MEK signaling pathways. J. Microbiol. Biotechnol..

[69] Barros M.P., Poppe S.C., Bondan E.F. (2014). Neuroprotective properties of the marine carotenoid astaxanthin and omega-3 fatty acids, and perspectives for the natural combination of both in krill oil. Nutrients.

[70] Nakamura T., Matsumoto J., Takamura Y., Ishii Y., Sasahara M., Ono T., Nishijo H. (2015). Relationships among parvalbumin-immunoreactive neuron density, phase-locked gamma oscillations, and autistic/schizophrenic symptoms in PDGFR-β knock-out and control mice. PLoS ONE.

[71] Iaccarino H.F., Singer A.C., Martorell A.J., Rudenko A., Gao F., Gillingham T.Z., Mathys H., Seo J., Kritskiy O., Abdurrob F. (2016). Gamma frequency entrainment attenuates amyloid load and modifies microglia. Nature.

[72] Hellwig S., Masuch A., Nestel S., Katzmarski N., Meyer-Luehmann M., Biber K. (2015). Forebrain microglia from wild-type but not adult 5xFAD mice prevent amyloid-β plaque formation in organotypic hippocampal slice cultures. Sci. Rep..

[73] Zhu X., Chen Y., Chen Q., Yang H., Xie X. (2018). Astaxanthin Promotes Nrf2/ARE Signaling to Alleviate Renal Fibronectin and Collagen IV Accumulation in Diabetic Rats. J. Diabetes Res..

[74] Jo C., Gundemir S., Pritchard S., Jin Y.N., Rahman I., Johnson G.V. (2014). Nrf2 reduces levels of phosphorylated tau protein by inducing autophagy adaptor protein NDP52. Nat. Commun..

[75] Hafez H.A., Kamel M.A., Osman M.Y., Osman H.M., Elblehi S.S., Mahmoud S.A. (2021). Ameliorative effects of astaxanthin on brain tissues of alzheimer’s disease-like model: Cross talk between neuronal-specific microRNA-124 and related pathways. Mol. Cell. Biochem..

[76] Shalash A.S., Hamid E., Elrassas H., Bahbah E.I., Mansour A.H., Mohamed H., Elbalkimy M. (2021). Non-motor symptoms in essential tremor, akinetic rigid and tremor-dominant subtypes of Parkinson’s disease. PLoS ONE.

[77] Elfil M., Bahbah E.I., Attia M.M., Eldokmak M., Koo B.B. (2021). Impact of Obstructive Sleep Apnea on Cognitive and Motor Functions in Parkinson’s Disease. Mov. Disord..

[78] Strickland D., Bertoni J.M. (2004). Parkinson’s prevalence estimated by a state registry. Mov. Disord. Off. J. Mov. Disord. Soc..

[79] Tysnes O.B., Storstein A. (2017). Epidemiology of Parkinson’s disease. J. Neural Transm..

[80] Archibald N., Miller N., Rochester L. (2013). Neurorehabilitation in Parkinson disease. Handb. Clin. Neurol..

[81] Shtilbans A., Henchcliffe C. (2012). Biomarkers in Parkinson’s disease: An update. Curr. Opin. Neurol..

[82] Kwan L.C., Whitehill T.L. (2011). Perception of Speech by Individuals with Parkinson’s Disease: A Review. Parkinsons Disease.

[83] Ge H., Yan Z., Zhu H., Zhao H. (2019). MiR-410 exerts neuroprotective effects in a cellular model of Parkinson’s disease induced by 6-hydroxydopamine via inhibiting the PTEN/AKT/mTOR signaling pathway. Exp. Mol. Pathol..

[84] Leggio L., Vivarelli S., L’Episcopo F., Tirolo C., Caniglia S., Testa N., Marchetti B., Iraci N. (2017). microRNAs in Parkinson’s Disease: From Pathogenesis to Novel Diagnostic and Therapeutic Approaches. Int. J. Mol. Sci..

[85] McMillan K.J., Murray T.K., Bengoa-Vergniory N., Cordero-Llana O., Cooper J., Buckley A., Wade-Martins R., Uney J.B., O’Neill M.J., Wong L.F. (2017). Loss of MicroRNA-7 Regulation Leads to α-Synuclein Accumulation and Dopaminergic Neuronal Loss In Vivo. Mol. Ther. J. Am. Soc. Gene Ther..

[86] Titze-de-Almeida R., Titze-de-Almeida S.S. (2018). miR-7 Replacement Therapy in Parkinson’s Disease. Curr. Gene Ther..

[87] Shen D.F., Qi H.P., Ma C., Chang M.X., Zhang W.N., Song R.R. (2020). Astaxanthin suppresses endoplasmic reticulum stress and protects against neuron damage in Parkinson’s disease by regulating miR-7/SNCA axis. Neurosci. Res..

[88] Grimmig B., Daly L., Subbarayan M., Hudson C., Williamson R., Nash K., Bickford P.C. (2018). Astaxanthin is neuroprotective in an aged mouse model of Parkinson’s disease. Oncotarget.

[89] Wang C.C., Shi H.H., Xu J., Yanagita T., Xue C.H., Zhang T.T., Wang Y.M. (2020). Docosahexaenoic acid-acylated astaxanthin ester exhibits superior performance over non-esterified astaxanthin in preventing behavioral deficits coupled with apoptosis in MPTP-induced mice with Parkinson’s disease. Food Funct..

[90] Wang X.J., Chen W., Fu X.T., Ma J.K., Wang M.H., Hou Y.J., Tian D.C., Fu X.Y., Fan C.D. (2018). Reversal of homocysteine-induced neurotoxicity in rat hippocampal neurons by astaxanthin: Evidences for mitochondrial dysfunction and signaling crosstalk. Cell Death Discov..

[91] Ye Q., Zhang X., Huang B., Zhu Y., Chen X. (2013). Astaxanthin suppresses MPP(+)-induced oxidative damage in PC12 cells through a Sp1/NR1 signaling pathway. Mar. Drugs.

[92] Ye Q., Huang B., Zhang X., Zhu Y., Chen X. (2012). Astaxanthin protects against MPP(+)-induced oxidative stress in PC12 cells via the HO-1/NOX2 axis. BMC Neurosci..

[93] Liu X., Shibata T., Hisaka S., Osawa T. (2009). Astaxanthin inhibits reactive oxygen species-mediated cellular toxicity in dopaminergic SH-SY5Y cells via mitochondria-targeted protective mechanism. Brain Res..

[94] Ikeda Y., Tsuji S., Satoh A., Ishikura M., Shirasawa T., Shimizu T. (2008). Protective effects of astaxanthin on 6-hydroxydopamine-induced apoptosis in human neuroblastoma SH-SY5Y cells. J. Neurochem..

[95] Finnerup N.B., Haroutounian S., Kamerman P., Baron R., Bennett D.L.H., Bouhassira D., Cruccu G., Freeman R., Hansson P., Nurmikko T. (2016). Neuropathic pain: An updated grading system for research and clinical practice. Pain.

[96] Kramer J.L., Minhas N.K., Jutzeler C.R., Erskine E.L., Liu L.J., Ramer M.S. (2017). Neuropathic pain following traumatic spinal cord injury: Models, measurement, and mechanisms. J. Neurosci. Res..

[97] Lampert A., Hains B.C., Waxman S.G. (2006). Upregulation of persistent and ramp sodium current in dorsal horn neurons after spinal cord injury. Exp. Brain Res..

[98] Naseri K., Saghaei E., Abbaszadeh F., Afhami M., Haeri A., Rahimi F., Jorjani M. (2013). Role of microglia and astrocyte in central pain syndrome following electrolytic lesion at the spinothalamic tract in rats. J. Mol. Neurosci. Mn.

[99] D’Angelo R., Morreale A., Donadio V., Boriani S., Maraldi N., Plazzi G., Liguori R. (2013). Neuropathic pain following spinal cord injury: What we know about mechanisms, assessment and management. Eur. Rev. Med. Pharmacol. Sci..

[100] Finnerup N.B., Otto M., McQuay H.J., Jensen T.S., Sindrup S.H. (2005). Algorithm for neuropathic pain treatment: An evidence based proposal. Pain.

[101] Gugliandolo E., D’amico R., Cordaro M., Fusco R., Siracusa R., Crupi R., Impellizzeri D., Cuzzocrea S., Di Paola R. (2018). Effect of PEA-OXA on neuropathic pain and functional recovery after sciatic nerve crush. J. Neuroinflamm..

[102] Sharma K., Sharma D., Sharma M., Sharma N., Bidve P., Prajapati N., Kalia K., Tiwari V. (2018). Astaxanthin ameliorates behavioral and biochemical alterations in in-vitro and in-vivo model of neuropathic pain. Neurosci. Lett..

[103] Jiang X., Yan Q., Liu F., Jing C., Ding L., Zhang L., Pang C. (2018). Chronic trans-astaxanthin treatment exerts antihyperalgesic effect and corrects co-morbid depressive like behaviors in mice with chronic pain. Neurosci. Lett..

[104] Fakhri S., Dargahi L., Abbaszadeh F., Jorjani M. (2019). Effects of astaxanthin on sensory-motor function in a compression model of spinal cord injury: Involvement of ERK and AKT signalling pathway. Eur. J. Pain.

[105] Fakhri S., Dargahi L., Abbaszadeh F., Jorjani M. (2018). Astaxanthin attenuates neuroinflammation contributed to the neuropathic pain and motor dysfunction following compression spinal cord injury. Brain Res. Bull..

[106] Marvizón J.C., McRoberts J.A., Ennes H.S., Song B., Wang X., Jinton L., Corneliussen B., Mayer E.A. (2002). Two N-methyl-D-aspartate receptors in rat dorsal root ganglia with different subunit composition and localization. J. Comp. Neurol..

[107] Gorman A.L., Yu C.G., Ruenes G.R., Daniels L., Yezierski R.P. (2001). Conditions affecting the onset, severity, and progression of a spontaneous pain-like behavior after excitotoxic spinal cord injury. J. Pain.

[108] Yu C.G., Fairbanks C.A., Wilcox G.L., Yezierski R.P. (2003). Effects of agmatine, interleukin-10, and cyclosporin on spontaneous pain behavior after excitotoxic spinal cord injury in rats. J. Pain.

[109] Hains B.C., Waxman S.G. (2007). Sodium channel expression and the molecular pathophysiology of pain after SCI. Prog. Brain Res..

[110] Ji R.R., Woolf C.J. (2001). Neuronal plasticity and signal transduction in nociceptive neurons: Implications for the initiation and maintenance of pathological pain. Neurobiol. Dis..

[111] Lerch J.K., Puga D.A., Bloom O., Popovich P.G. (2014). Glucocorticoids and macrophage migration inhibitory factor (MIF) are neuroendocrine modulators of inflammation and neuropathic pain after spinal cord injury. Semin. Immunol..

[112] Baastrup C., Finnerup N.B. (2008). Pharmacological management of neuropathic pain following spinal cord injury. CNS Drugs.

[113] Fakhri S., Aneva I.Y., Farzaei M.H., Sobarzo-Sánchez E. (2019). The Neuroprotective Effects of Astaxanthin: Therapeutic Targets and Clinical Perspective. Molecules.

[114] Yamagishi R., Aihara M. (2014). Neuroprotective effect of astaxanthin against rat retinal ganglion cell death under various stresses that induce apoptosis and necrosis. Mol. Vis..

[115] Ikonomidou C., Bosch F., Miksa M., Bittigau P., Vöckler J., Dikranian K., Tenkova T.I., Stefovska V., Turski L., Olney J.W. (1999). Blockade of NMDA receptors and apoptotic neurodegeneration in the developing brain. Science.

[116] Alexander J.K., Cox G.M., Tian J.B., Zha A.M., Wei P., Kigerl K.A., Reddy M.K., Dagia N.M., Sielecki T., Zhu M.X. (2012). Macrophage migration inhibitory factor (MIF) is essential for inflammatory and neuropathic pain and enhances pain in response to stress. Exp. Neurol..

[117] Islam M.S., Kanak F., Iqbal M.A., Islam K.F., Al-Mamun A., Uddin M.S. (2018). Analyzing the Status of the Autism Spectrum Disorder Amid Children with Intellectual Disabilities in Bangladesh. Biomed. Pharmacol. J..

[118] Boyle C.A., Boulet S., Schieve L.A., Cohen R.A., Blumberg S.J., Yeargin-Allsopp M., Visser S., Kogan M.D. (2011). Trends in the prevalence of developmental disabilities in US children, 1997-2008. Pediatrics.

[119] Kern J.K., Geier D.A., Sykes L.K., Geier M.R. (2013). Evidence of neurodegeneration in autism spectrum disorder. Transl. Neurodegener..

[120] Kemper T.L., Bauman M. (1998). Neuropathology of infantile autism. J. Neuropathol. Exp. Neurol..

[121] Lee M., Martin-Ruiz C., Graham A., Court J., Jaros E., Perry R., Iversen P., Bauman M., Perry E. (2002). Nicotinic receptor abnormalities in the cerebellar cortex in autism. Brain A J. Neurol..

[122] Courchesne E., Pierce K., Schumann C.M., Redcay E., Buckwalter J.A., Kennedy D.P., Morgan J. (2007). Mapping early brain development in autism. Neuron.

[123] Li X., Chauhan A., Sheikh A.M., Patil S., Chauhan V., Li X.M., Ji L., Brown T., Malik M. (2009). Elevated immune response in the brain of autistic patients. J. Neuroimmunol..

[124] Sabit H., Tombuloglu H., Rehman S., Almandil N.B., Cevik E., Abdel-Ghany S., Rashwan S., Abasiyanik M.F., Yee Waye M.M. (2021). Gut microbiota metabolites in autistic children: An epigenetic perspective. Heliyon.

[125] Granot E., Kohen R. (2004). Oxidative stress in childhood--in health and disease states. Clin. Nutr..

[126] Evans T.A., Siedlak S.L., Lu L., Fu X., Wang Z., McGinnis W.R., Fakhoury E., Castellani R.J., Hazen S.L., Walsh W.J. (2008). The autistic phenotype exhibits a remarkably localized modification of brain protein by products of free radical-induced lipid oxidation. Am. J. Biochem. Biotechnol..

[127] Sajdel-Sulkowska E., Lipinski B., Windom H., Audhya T., McGinnis W. (2008). Oxidative stress in autism: Elevated cerebellar 3-nitrotyrosine levels. Am. J. Biochem. Biotechnol..

[128] Sajdel-Sulkowska E.M., Xu M., McGinnis W., Koibuchi N. (2011). Brain region-specific changes in oxidative stress and neurotrophin levels in autism spectrum disorders (ASD). Cerebellum.

[129] Krajcovicova-Kudlackova M., Valachovicova M., Mislanova C., Hudecova Z., Sustrova M., Ostatnikova D. (2009). Plasma concentrations of selected antioxidants in autistic children and adolescents. Bratisl Lek Listy.

[130] Ornoy A., Weinstein-Fudim L., Ergaz Z. (2019). Prevention or Amelioration of Autism-Like Symptoms in Animal Models: Will it Bring Us Closer to Treating Human ASD? Int. J. Mol. Sci..

[131] Lyu Y., Wu L., Wang F., Shen X., Lin D. (2018). Carotenoid supplementation and retinoic acid in immunoglobulin A regulation of the gut microbiota dysbiosis. Exp. Biol. Med. (Maywood).

[132] Fernández M.J.F., Valero-Cases E., Rincon-Frutos L. (2019). Food Components with the Potential to be Used in the Therapeutic Approach of Mental Diseases. Curr. Pharm. Biotechnol..

[133] Al-Amin M.M., Rahman M.M., Khan F.R., Zaman F., Mahmud Reza H. (2015). Astaxanthin improves behavioral disorder and oxidative stress in prenatal valproic acid-induced mice model of autism. Behav. Brain Res..

[134] Xue Y., Qu Z., Fu J., Zhen J., Wang W., Cai Y., Wang W. (2017). The protective effect of astaxanthin on learning and memory deficits and oxidative stress in a mouse model of repeated cerebral ischemia/reperfusion. Brain Res. Bull..

[135] Wang Y.L., Zhu X.L., Sun M.H., Dang Y.K. (2019). Effects of astaxanthin onaxonal regeneration via cAMP/PKA signaling pathway in mice with focal cerebral infarction. Eur. Rev. Med. Pharmacol. Sci..

[136] Zhang J., Ding C., Zhang S., Xu Y. (2020). Neuroprotective effects of astaxanthin against oxygen and glucose deprivation damage via the PI3K/Akt/GSK3β/Nrf2 signalling pathway in vitro. J. Cell. Mol. Med..

[137] Nai Y., Liu H., Bi X., Gao H., Ren C. (2018). Protective effect of astaxanthin on acute cerebral infarction in rats. Hum. Exp. Toxicol..

[138] Cakir E., Cakir U., Tayman C., Turkmenoglu T.T., Gonel A., Turan I.O. (2020). Favorable Effects of Astaxanthin on Brain Damage due to Ischemia- Reperfusion Injury. Comb. Chem. High Throughput Screen..

[139] Carelli S., Giallongo T., Gombalova Z., Rey F., Gorio M.C.F., Mazza M., Di Giulio A.M. (2018). Counteracting neuroinflammation in experimental Parkinson’s disease favors recovery of function: Effects of Er-NPCs administration. J. Neuroinflamm..

[140] Stewart J.S., Lignell A., Pettersson A., Elfving E., Soni M.G. (2008). Safety assessment of astaxanthin-rich microalgae biomass: Acute and subchronic toxicity studies in rats. Food Chem. Toxicol. Int. J. Publ. Br. Ind. Biol. Res. Assoc..

[141] Hussein G., Nakamura M., Zhao Q., Iguchi T., Goto H., Sankawa U., Watanabe H. (2005). Antihypertensive and neuroprotective effects of astaxanthin in experimental animals. Biol. Pharm. Bull..

[142] EFSA Panel on Dietetic Products N. (2014). Allergies. Scientific Opinion on the safety of astaxanthin-rich ingredients (AstaREAL A1010 and AstaREAL L10) as novel food ingredients. EFSA J..

[143] Additives E.P.O., Feed P.O.S.U.I.A. (2014). Scientific Opinion on the safety and efficacy of synthetic astaxanthin as feed additive for salmon and trout, other fish, ornamental fish, crustaceans and ornamental birds. EFSA J..

[144] Imai A., Oda Y., Ito N., Seki S., Nakagawa K., Miyazawa T., Ueda F. (2018). Effects of Dietary Supplementation of Astaxanthin and Sesamin on Daily Fatigue: A Randomized, Double-Blind, Placebo-Controlled, Two-Way Crossover Study. Nutrients.

[145] Mashhadi N.S., Zakerkish M., Mohammadiasl J., Zarei M., Mohammadshahi M., Haghighizadeh M.H. (2018). Astaxanthin improves glucose metabolism and reduces blood pressure in patients with type 2 diabetes mellitus. Asia Pac. J. Clin. Nutr..

[146] Brendler T., Williamson E.M. (2019). Astaxanthin: How much is too much? A safety review. Phytother. Res..

[147] Kajita M., Kato T., Yoshimoto T., Masuda K. (2010). Study on the safety of high-dose administration of astaxanthin. Folia Jpn. Ophthalmol. Clin..

[148] Kupcinskas L., Lafolie P., Lignell A., Kiudelis G., Jonaitis L., Adamonis K., Andersen L.P., Wadström T. (2008). Efficacy of the natural antioxidant astaxanthin in the treatment of functional dyspepsia in patients with or without Helicobacter pylori infection: A prospective, randomized, double blind, and placebo-controlled study. Phytomedicine Int. J. Phytother. Phytopharm..

[149] Katsumata T., Ishibashi T., Kyle D. (2014). A sub-chronic toxicity evaluation of a natural astaxanthin-rich carotenoid extract of Paracoccus carotinifaciens in rats. Toxicol. Rep..

